# Fatal outcome of congenital aortopulmonary window with patent ductus arteriosus complicating pregnancy

**DOI:** 10.4322/acr.2021.265

**Published:** 2021-04-19

**Authors:** Balamurugan Thirunavukkarasu, Lijanthung S. Kithan, Nikhil Kumar, Arihant Jain, Amanjit Bal

**Affiliations:** 1 Post Graduate Institute of Medical Education & Research (PGIMER), Departments of Histopathology, Chandigarh, India; 2 Post Graduate Institute of Medical Education & Research (PGIMER), Departments of Internal Medicine, Chandigarh, India

**Keywords:** Aortopulmonary window, Heart defects, Congenital, Hypertension, Pulmonary, Autopsy

## Abstract

Aortopulmonary window (APW) is a rare congenital heart defect with abnormal communication between the ascending aorta and the pulmonary trunk with two separate semilunar valves. We present an autopsy case report wherein a young primigravida woman presented with progressive breathlessness and central cyanosis at 21 weeks of gestation. Echocardiography performed in the emergency room revealed elevated right-sided cardiac pressures suggestive of severe pulmonary hypertension; however, no structural cardiac defect was discernible. The patient succumbed to congestive cardiac failure and progressive hypoxia within 5 days of hospitalization. The autopsy revealed a Type I aortopulmonary window (2 cm) with patent ductus arteriosus. The lungs showed changes of severe pulmonary hypertension with superadded bronchopneumonia. This report underscores a rare presentation of APW, undiagnosed until pregnancy, leading to the Eisenmenger syndrome and death.

## INTRODUCTION

Aortopulmonary window (APW) is a rare congenital heart defect that accounts for 0.1-0.2% of all congenital heart defects.^1^ ^-^ ^3^ It is characterized by abnormal communication between the ascending aorta and pulmonary trunk with two separate aortic and pulmonary valves producing a left-to-right shunt. The presentation time can be variable, ranging from the prenatal period to as late as the seventh decade.^4^ Surgical correction at an early stage can prevent the development of irreversible pulmonary hypertension (PHT) and Eisenmenger syndrome.^5^ We describe an adult case of APW associated with patent ductus arteriosus who first presented at 21 weeks of gestation with cyanosis and rapidly succumbed to her illness.

## CASE REPORT

A 23-year-old primigravida with no known previous illness presented at 21 weeks of gestation with progressive new-onset shortness of breath, dizziness on exertion, and cyanosis for 3 months. Examination revealed a respiratory rate of 32/min, a heart rate of 110/min, and SpO2 of 27% on room air. She had bilateral, symmetrical, Grade 2 clubbing fingers and toes along with cyanosis on lips, fingers, and nails that persisted despite oxygen supplementation. There was a parasternal heave with loud P2. Investigations revealed hemoglobin of 13.7g/dl (reference range [RR]; 12-15 g/dl), leukocyte count of 18x10^9^/L (RR; 4.5-11 x10^9^/L) and platelet count of 67x10^9^/L (RR; 150-400 x10^9^/L) and chest x-ray showed cardiomegaly. Transthoracic echocardiogram showed a dilated right atrium (RA) and right ventricle (RV) with moderate tricuspid regurgitation. The main pulmonary artery was dilated to 23mm, and the pulmonary acceleration time was 59ms indicating severe pulmonary hypertension (<60ms – Severe PHT). No definite atrial septal defect, ventricular septal defect, or other anomalies were seen. A possibility of primary pulmonary hypertension was kept, and the patient was started on Sildenafil 40mg three times a day. The patient had an intrauterine death with spontaneous labor and expulsion of the fetus on 2nd day of hospitalization. Despite mechanical ventilation, the patient had persistent hypoxia and refractory shock, to which she succumbed on the third day of hospitalization. A partial autopsy was performed after written consent.

## AUTOPSY FINDINGS

**Figure 1:**
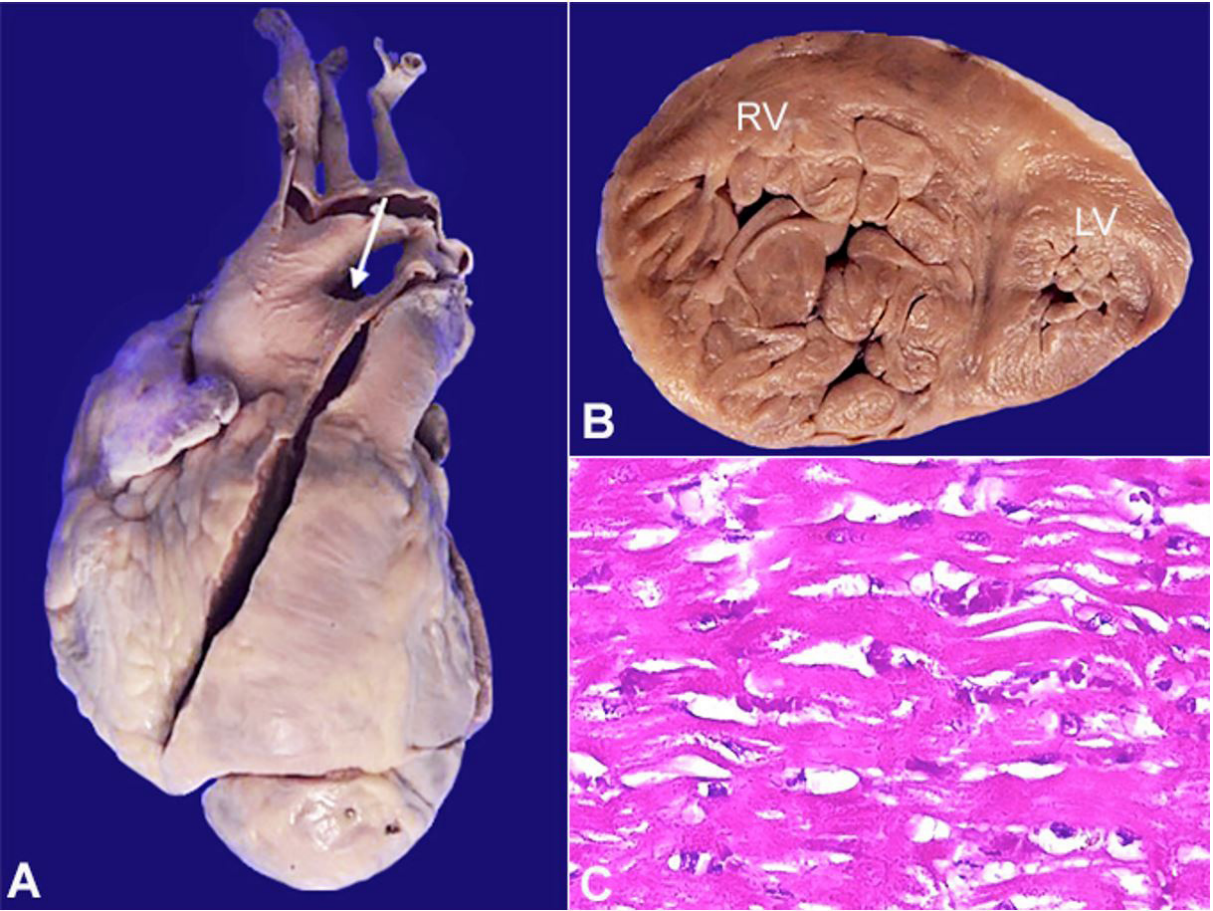
**A –** External view: Right ventricle forming the apex indicating severe enlargement. White arrow – Lack of separation between the aorta and pulmonary trunk; **B –** Apical slice indicating severe right ventricular hypertrophy; **C –** Microscopy showing hypertrophic cardiac myocytes.

**Figure 2:**
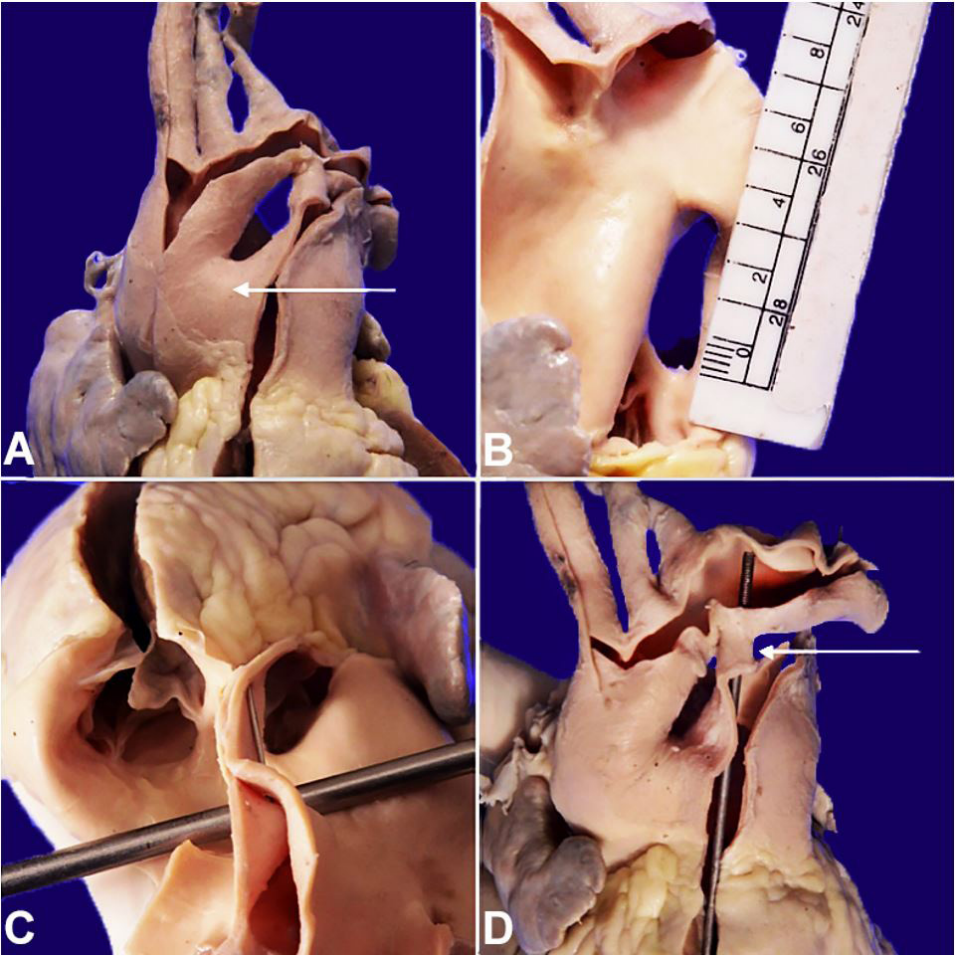
Aortopulmonary window. **A** – Anterior view (arrow pointed); **B –** Right lateral view showing Type 1 defect; **C –** Superior view with the probe placed in the defect and showing intact semilunar valves; **D** – Patent ductus arteriosus (Probe placed in situ with an arrow pointed).

On the gross examination, the heart weighed 260 grams (mean RR; 276 g), and the apex was rounded and mainly formed by the right ventricle (Figure 1A). There was a right atrial dilatation with marked right ventricular hypertrophy (wall thickness of 1.2 cm [RR 0.35-0.40 cm]) (Figure 1B). A large aortopulmonary window connecting the main pulmonary artery and the ascending aorta (Type I) in the proximal region, measuring 2 cm in the greatest dimension, was noted (Figure 2AC). In addition, there was a patent ductus arteriosus (Figure 2D). The left atrium and atrioventricular valves were unremarkable. The left ventricular wall thickness was 1.3 cm (RR; 1.05-1.25 cm). The pulmonary and aortic valves were separate and normally developed (Figure 2C). No atrial or ventricular septal defect was seen. The microscopic examination from the right ventricle showed cytoplasmic eosinophilia and mild anisonucleosis, confirming right ventricular hypertrophy (Figure 1C).

**Figure 3:**
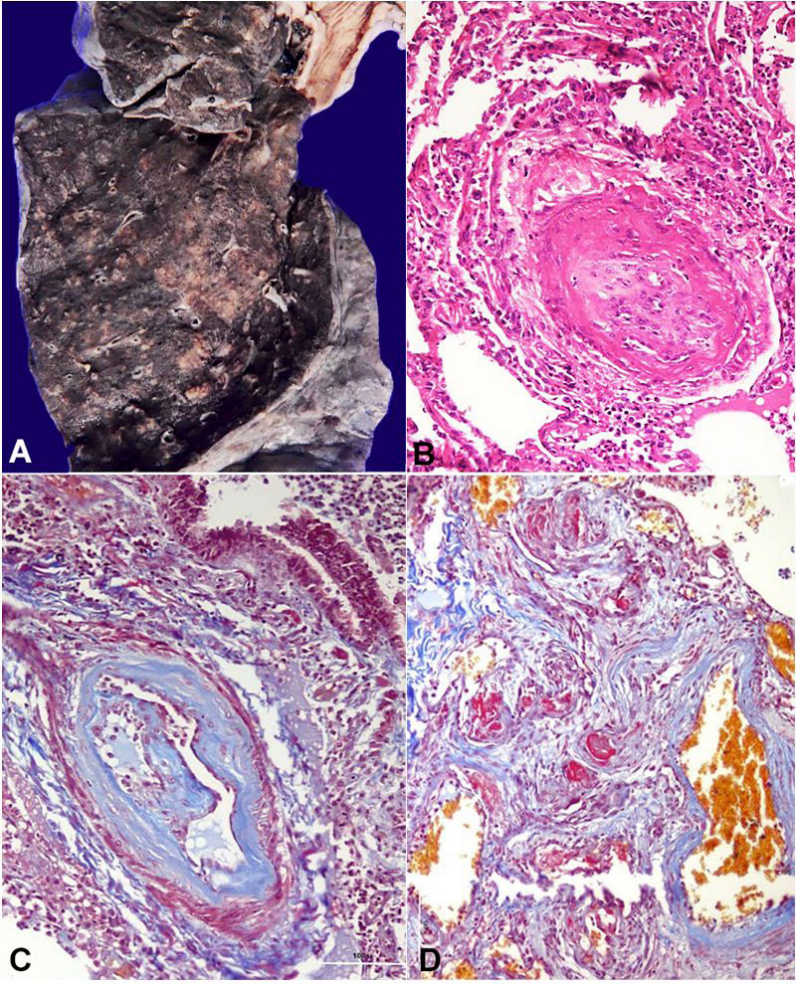
**A –** Cut surface of lung shows prominent bronchovascular marking with multiple small nodules; **B –** Pulmonary artery hypertension in the form of myointimal proliferation with obliteration of lumen (H&E, 200X); **C –** Masson trichrome stain (200X); **D –** Plexiform lesion (Masson trichrome, 200X).

Both lungs were heavy, weighing 800 g (mean RR; 730 g), and the cut surface showed prominent broncho-vascular markings. There were multiple bronchiolocentric nodules (5mm) predominantly seen in the lower lobes (Figure 3A). Multiple sections showed a spectrum of changes related to pulmonary hypertension. The pre-acinar and intra-acinar pulmonary artery branches showed eccentric myointimal proliferation with complete occlusion of the lumen. In addition, the plexiform lesions with intraluminal fibrin deposition were occasionally seen (Figure 3BD). Sections studied from the nodules showed bronchopneumonia composed of neutrophilic infiltrate in the alveoli.

The special stains did not highlight any bacterial or fungal profiles. No evidence of amniotic fluid embolism or pulmonary embolism was noted. Sections from the liver, spleen, and pancreas were unremarkable.

The final autopsy diagnosis was a Type 1 aortopulmonary window with patent ductus arteriosus. The lung showed changes of severe pulmonary arteriopathy with superadded bronchopneumonia. The fetal autopsy was performed but did not show any congenital malformations, including cardiac defect.

## DISCUSSION

The aortopulmonary window arises due to embryonic failure of the fusion of two opposing conotruncal ridge or spiral septum, which separates the truncus arteriosus into the aorta and pulmonary artery. The defect causes left to right shunt and was first described by John Elliotson.^6^ Few classification schemes exist for APW based on the location of the defect.^7^ ^,^ ^8^ Mori et al.^7^ have classified APW as proximal or type I, distal or type II, and total or type III defect. Our case had an oval defect just above the semilunar valve (proximal defect), placing it in Type I category.^7^ Classification by the Society of Thoracic Surgeons is another common classification system, which is followed.^8^ Associations with other cardiac anomalies have been described in nearly one-third to half of cases, of which the most common is interrupted aortic arch.^3^ ^,^ ^9^ Other associated anomalies include patent ductus arteriosus, coronary artery anomalies, ventricular septal defect, atrial septal defect, tetralogy of Fallot, and transposition of great arteries.^10^ ^-^ ^12^ In a series of 42 pediatric cases of APW, 16 cases (38%) had associated patent ductus arteriosus.^11^ APW with PDA first presenting in adult has been rarely reported in only one case report, to the best of our knowledge.^13^

The usual course of APW varies according to the size of the defect and its associated anomaly. A large defect can lead to the left-right shunt causing cardiac failure, pulmonary hypertension, Eisenmenger syndrome, and death in infancy or early childhood.^13^ Timely diagnosis of APW by prenatal echocardiography can facilitate timely referral for surgical repair prior to the development of irreversible Eisenmenger syndrome that has high mortality up to 40%.^14^ Undiagnosed cases may have poor weight gain and recurrent respiratory illness. Cardiovascular diseases have been reported in 1-4% of pregnancies, of which few can lead to severe morbidity and mortality.^15^ Clinical diagnosis of the aortopulmonary window should be suspected in adult patients with pulmonary hypertension after excluding the other causes. 2D echocardiography has a variable sensitivity of 37-57% in APW, depending on factors like the type of instrument, operator experience, type of anomaly (Proximal > Distal defect), and the presence of pulmonary hypertension. Our case had very high PA pressure, and therefore though there were an anatomic APW and PDA, but there would have been no flow across the shunt and, therefore, not visible on echocardiography. Like the current case, APW may be missed at the initial presentation, unless there is a high threshold of suspicion.^16^ ^,^ ^17^ The diagnosis by 2DE should be made by visualizing the aortopulmonary septum in two or more different planes with the best visualization in a high parasternal short-axis view. Doppler and color flow mapping add to the sensitivity of echocardiography. Transesophageal echocardiography and magnetic resonance angiography may be useful in a difficult case. Though the role of non-invasive techniques have increased drastically in recent times, cardiac catheterization can definitely help in cases of complex anomalies and in cases with established pulmonary hypertension.^13^ ^,^ ^18^

The index patient survived into adulthood without any significant illness. Survival into adulthood with such a lethal defect is an intriguing and enigmatic phenomenon. The influencing factors include the patient’s physiological, anatomical, and psychological adaptation, associated co-morbidities, cardiac remodeling, and other associated cardiac anomalies.^19^ These patients develop Eisenmenger at a very early age of less than 5 years. The patient might not become symptomatic ever due to multiple reasons like non-regression of high infantile PA pressures. Once they develop Eisenmenger because of reduced L→R shunt, they become symptomatically better with respect to heart failure symptoms. Pregnancy-associated hemodynamic changes include increased plasma volume, cardiac output, dilutional anemia, and reduction in systemic vascular resistance (SVR).^20^ Decreased SVR during pregnancy leads to an increase in right to left shunt, subsequently leading to reduced pulmonary perfusion and hypoxia. Hence, the mortality of this patient is due to right ventricular failure due to very high PA pressures, which got accentuated because of pneumonia, in our case.

There is scarce literature regarding the feto-maternal outcomes of pregnancy with uncorrected APW. To date, four cases have been described to the best of our knowledge and are summarized in Table 1. Two cases had an uneventful pregnancy and postpartum period.^4^ ^,^ ^21^ One case had a clinical profile similar to the present case; however, the patient survived the pregnancy and delivered the child by a cesarean section.^22^ In another case, the pregnancy was terminated, and the patient expired years later.^19^

**Table 1:** Adult cases of Aortopulmonary window – related to or post-pregnancy

**Author**	**Age of diagnosis (years)**	**Mode of diagnosis**	**Maternal outcome**	**Fetal outcome**
Su-Mei and Ju-Le^4^	40	Echocardiography	Asymptomatic for 50 years; Died at 60 years of age due to biventricular failure;	Delivered three children during her lifetime which was uneventful
Aggarwal et al.^21^	25	CECT chest#	Diagnosed immediate postpartum – alive	Successfully completed 1^st^ pregnancy
Kose et al.^22^	27	Cardiac catheterization	Asymptomatic since birth; Diagnosed at 27 weeks of gestation;	Successfully delivered baby at 35^th^ week
Niles and Schmidt^19^	39	Cardiac catheterization and later autopsy	Symptomatic since early childhood; Died at 46 years of age	Terminated pregnancy at age of 20 years
Current case	23	Autopsy	Symptomatic during 2nd trimester of pregnancy – Died due to cardiac failure and Eisenmenger syndrome	Spontaneous abortion

# CECT: Contrast-enhanced computed tomography.

Treatment of APW depends on the size of the defect, associated anomalies such as the interrupted aortic arch, and anomalous origin of coronary arteries. The defect can be repaired via an incision in the window, through the aorta, or through the pulmonary artery and closed with an appropriately sized patch. Catheter-based closure is appropriate in small-sized defects.^9^

## CONCLUSION

This report highlights an autopsy-based demonstration of an undiagnosed case of the aortopulmonary window with patent ductus arteriosus in its untreated natural course. In her early twenties, this adult patient had established pulmonary hypertension with a reversal of shunt that worsened due to the hemodynamic alterations of pregnancy and superadded pneumonia. A high index of suspicion can lead to timely diagnosis and surgical management.
